# MobiDB: 10 years of intrinsically disordered proteins

**DOI:** 10.1093/nar/gkac1065

**Published:** 2022-11-23

**Authors:** Damiano Piovesan, Alessio Del Conte, Damiano Clementel, Alexander Miguel Monzon, Martina Bevilacqua, Maria Cristina Aspromonte, Javier A Iserte, Fernando E Orti, Cristina Marino-Buslje, Silvio C E Tosatto

**Affiliations:** Department of Biomedical Sciences, University of Padova, Padova, Italy; Department of Biomedical Sciences, University of Padova, Padova, Italy; Department of Biomedical Sciences, University of Padova, Padova, Italy; Department of Information Engineering, University of Padova, Padova, Italy; Department of Biomedical Sciences, University of Padova, Padova, Italy; Department of Biomedical Sciences, University of Padova, Padova, Italy; Bioinformatics Unit, Fundación Instituto Leloir, Buenos Aires, Argentina; Bioinformatics Unit, Fundación Instituto Leloir, Buenos Aires, Argentina; Bioinformatics Unit, Fundación Instituto Leloir, Buenos Aires, Argentina; Department of Biomedical Sciences, University of Padova, Padova, Italy

## Abstract

The MobiDB database (URL: https://mobidb.org/) is a knowledge base of intrinsically disordered proteins. MobiDB aggregates disorder annotations derived from the literature and from experimental evidence along with predictions for all known protein sequences. MobiDB generates new knowledge and captures the functional significance of disordered regions by processing and combining complementary sources of information. Since its first release 10 years ago, the MobiDB database has evolved in order to improve the quality and coverage of protein disorder annotations and its accessibility. MobiDB has now reached its maturity in terms of data standardization and visualization. Here, we present a new release which focuses on the optimization of user experience and database content. The major advances compared to the previous version are the integration of AlphaFoldDB predictions and the re-implementation of the homology transfer pipeline, which expands manually curated annotations by two orders of magnitude. Finally, the entry page has been restyled in order to provide an overview of the available annotations along with two separate views that highlight structural disorder evidence and functions associated with different binding modes.

## INTRODUCTION

Intrinsically disordered proteins (IDPs) and regions (IDRs) are characterized by the lack of a fixed three dimensional structure, they are generally more extended and exhibit an extreme dynamic behavior. Many functions of IDRs, such as entropic springs, flexible linkers or spacers are directly associated with their structural attributes (1,2). Main IDP functions are to form protein–protein and protein–DNA interactions (3), as well as their role in liquid–liquid phase separation (LLPS) (4). The majority of these interactions are provided by electrostatic forces and are entropy-driven with the formation of fuzzy complexes, where the folding energy of the interaction (folding upon binding) is compensated by an increase of structural heterogeneity of the rest of the protein (5,6). IDPs evolved to be versatile in terms of binding, yet they display very high specificity and low affinity, providing the cell with an effective tool to encode and develop transient interactions typical of signaling pathways. IDPs are highly regulated, offering an ideal platform to host targeted post-translational modification sites (7). In higher organisms IDPs are central in the regulation of the cell cycle and transcription processes (3). Viruses, instead, exploit IDRs to compress a high amount of functional information in a short genome and to evolve new hijacking mechanisms quickly (8).

Biological databases play a central role in accelerating biological discovery, making experimental information accessible in a standardized and structured way (9). However, difficulties in IDP expression, purification and structural characterization hamper experimental characterization and assigning functional modules to dynamic conformational ensembles presents a technical problem for a database. Available data is scattered across different specialized resources that are focused on different, often subtle, functional/structural aspects (10). MobiDB tackles this problem by aggregating, processing and visualizing primary data, and creates added value by providing an interpretation of the contained information.

**Figure 1.**
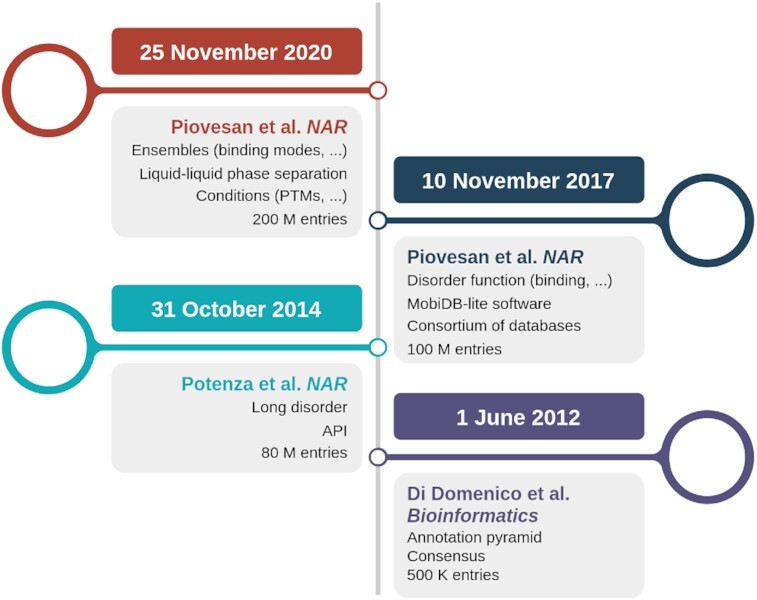
Ten years of MobiDB. Major achievements of MobiDB as described in the corresponding publications.

MobiDB celebrates 10 years of active development since it was first published in 2012 (11). The MobiDB roadmap is shown in Figure 1. From the beginning, MobiDB implemented the annotation pyramid concept and a set of rules to combine complementary information into consensus tracks. The intuition was to combine manually curated databases, experimentally derived information and state-of-the-art predictions, initially provided just by ESpritz (12) and IUPred (13). This expanded the annotation coverage to the entire Swiss-Prot, ca. 500 000 proteins, and at the same time acknowledged different levels of information quality, i.e. those from DisProt and PDB missing residues. The recognition of MobiDB as the reference for disorder annotations, became more clear from its second release in 2014 (14). It started to provide annotations for the entire UniProtKB, ca. 80 million sequences, and was cross-linked in the UniProtKB website. Notably, MobiDB started to exploit Web 2.0 functionalities by implementing a RESTful API for programmatic access based on a full-stack JavaScript server and website. The other big intuition that characterized all following releases of MobiDB since 2018 (15), was the addition of the function dimension to the annotation pyramid with a focus on binding. The ‘linear interacting peptide’ (LIP) term was coined as a container for all functional subcategories of binding and intermolecular interactions in PDB complexes, as provided by the RING software (16), were included. In the same release, along with an expansion of the variety of the integrated resources, MobiDB started to encapsulate all sequence based predictors in the MobiDB-lite package (17,18). MobiDB-lite was integrated into InterProScan (19), in order to guarantee a complete synchronization with major core data resources such as UniProtKB, InterPro (20) and PDBe-KB (21). In 2021, MobiDB (version 4 (22)) enriched the LIP concept by introducing ‘binding modes’ categories to better describe the specificity of different interaction complexes. Annotations of binding modes are provided by the FuzDB database that curates fuzzy complexes, i.e. those complexes that remain disordered in the bound state, and by a new in-house pipeline that can detect conditional folding associated to binding events by comparing PDB structures of the same protein complex in different binding forms, as described here (23). In the same release posttranslational modifications (PTMs) pulled from UniProtKB were made available on top of the feature viewer to compare structural states and conditional folding with PTM regulation.

In this article, we present the latest innovations introduced in MobiDB. First, we introduced AlphaFold predictions (24) which have been found to correlate very well with experimental disorder and with state-of-the art disorder predictors according to CAID assessment (25,26). AlphaFold predictions are also used to highlight positional annotations directly in protein structure whenever experimental coordinates are not available from the PDB. The second advance of MobiDB is the development of a new pipeline to transfer curated annotations based on homology inference, which increased the number of highly specific annotations by two orders of magnitude. Minor enhancements include the integration of the PhaSepDB (27) for liquid-liquid phase separation proteins and a refinement of the look and feel and user experience of the website, in particular for the entry pages.

## DATABASE CONTENT

### The annotation pyramid

MobiDB has been developed to serve both experimental scientists, interested in comprehensive information of single protein systems, as well as bioinformaticians, who seek large homogeneous collections of proteins sharing the same features to build statistical classifiers. In order to make its content more accessible to both scientific communities, MobiDB adopted the concept of ‘annotation pyramid’. The height of the pyramid represents the annotation quality while the horizontal axis is the coverage of known proteomes. Also, the MobiDB pyramid is staired to indicate different levels of evidence. In Table 1 are reported the number of entries along with the type and source of information for the four levels of the MobiDB pyramid. For each level and feature, MobiDB reports consensus annotations which are combined according to different sets of rules. The complete description of features, sources and consensus strategies are now available in a controlled vocabulary (see controlled vocabulary paragraph).

**Table 1.** Number of entries and annotation source for the four levels of the MobiDB data pyramid (MobiDB release 2022_07). For software sources, the input is indicated in parenthesis. (*) The FLIPPER repository includes MOBI (38) and additional in-house processing scripts to calculate disorder and binding modes features, respectively. (^) The full list of available software integrated into MobiDB-lite is provided in (15)

Evidence (size)	Feature	Source
Curated (4600)	Disorder, LIPs	DisProt (29)
	Disorder, LIPs	IDEAL (30)
	Disorder	Swiss-Prot / UniProtKB (28)
	LIPs	MFIB (31)
	LIPs	DIBS (32)
	LIPs	ELM (33)
	Binding modes	FuzDB (34)
	Conformational diversity	CoDNaS (35)
	LLPS	PhaSePro (36)
	LLPS	PhaSepDB (27)
Derived (59 076)	Disorder, LIPs, Binding modes	* FLIPPER (PDB structures) (37)
	Inter chain interactions	RING (PDB structures) (16)
Homology (458 167)	All curated features	In-house pipeline (curated and UniProtKB sequences)
Predicted (>200 M)	Disorder, LIPs, low complexity, compositional bias, secondary structure, structural rigidity	^ MobiDB-Lite (UniProtKB sequences) (17,18)
	Disorder, LIPs	AlphaFold-disorder (AlphaFoldDB) (26)

Curated annotations are pulled from the corresponding databases processing the data and checking their consistency. Curated entries are also used as input to infer homology and project their annotation to the rest of UniProtKB sequences (28). Derived annotations are extracted from PDB structures while sequence-based predictions are calculated with MobiDB-lite (18) which encapsulate a number of complementary predictors. The subset of disorder features provided by MobiDB-lite are the same provided by InterProScan (19) which propagates its predictions onto several other EBI resources like UniProtKB, InterPro (20) and PDBe-KB (21).

### Homology transfer

In the current version of MobiDB, manually curated annotations are transferred to other proteins based on homology inference (see Table 2). The search for homologous regions is performed starting from a full sequence BLAST alignment against the entire UniProtKB database and applying a filtering procedure in order to minimize the number of false positive instances.

**Table 2.** Number of entries before and after the annotation expansion provided by the homology pipeline (MobiDB release 2022_07). Expansion column indicates the number of homologous proteins divided by the number of curated entries

Feature	Source	Curated	Homologous	Expansion
Disorder	DisProt	2235	247 953	110.9
	IDEAL	974	105 089	107.9
	Swiss-Prot	194	7175	37.0
	Total	3063	340 550	111.2
Linear interacting peptide	DisProt	838	92 810	110.8
	DIBS	498	59 888	120.3
	MFIB	246	30 693	124.8
	IDEAL	216	30 202	139.8
	ELM	75	5452	72.7
	Total	1514	174 013	114.9
Phase separation	PhaSepDB	326	18 023	55.3
	PhaSePro	112	13 029	116.3
	Total	373	22 318	59.8
Fuzzy complexes	FuzDB	328	44 114	134.5
Conformational diversity	CoDNaS	1073	126 169	117.6

Alignments are performed starting from the full sequences in order to discard non significant matches. The pipeline starts from full sequence alignments but focuses only on alignment fragments corresponding to manually curated regions in MobiDB. The annotation is transferred when very stringent sequence similarity constraints are fulfilled. Specifically, the alignment fragment must cover the 90% of the query sequence (annotated region), gaps must not exceed the 20% of the length of the alignment and the subject (homologous region) must be 80% identical to the query region. In the case of multiple regions being identified on the same target protein, in order to remove overlaps, a greedy algorithm which prioritizes longer regions, is applied. Despite an expansion of two orders of magnitude, the homology transfer for low complexity regions is limited as they are masked by BLAST by default.

## NEW FEATURES

### AlphaFold and conditional disorder

**Figure 2.**
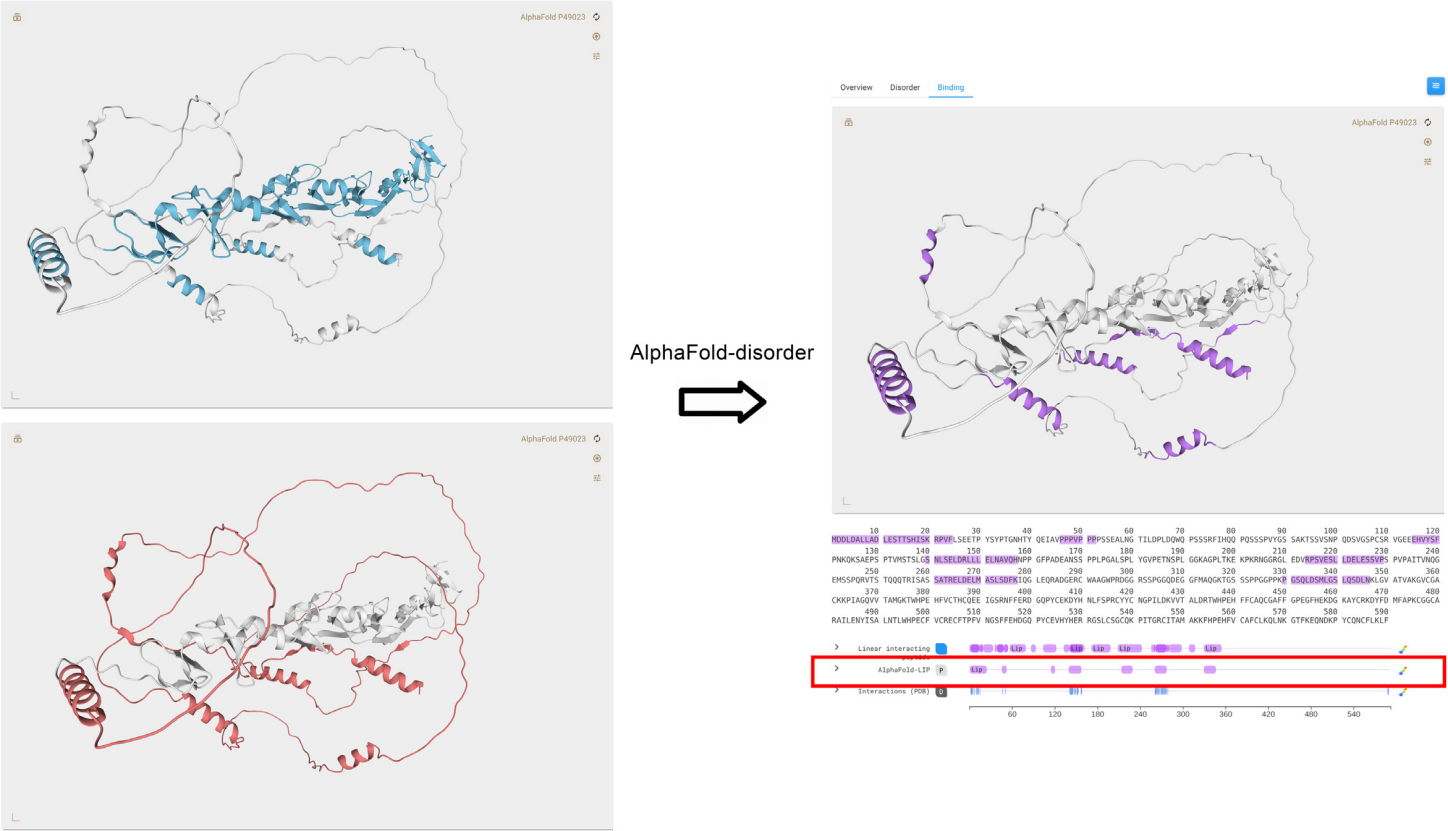
Conditional disorder in MobiDB. MobiDB entry page of the human Paxillin protein, accession P49023. On the left, the ‘AlphaFold-structure (pLDDT)’ (top) and ‘AlphaFold-disorder (RSA)’ (bottom) features, representing high-confidence well-structured regions (cyan) and exposed residues (red), respectively. On the right, the predicted LIPs (violet), resulting from the intersection between the two sets of residues highlighted on the left. The screenshots are taken from the ‘Disorder’ tab (left) and from the ‘Binding’ tab (right) of the entry page. Residue annotations are calculated with the AlphaFold-disorder script (26) applied to AlphaFoldDB predictions.

AlphaFold-2 is the most accurate predictor of protein structures (24) that has been proven to be also effective in identifying intrinsically disordered proteins (26). In MobiDB, AlphaFold predictions are processed in order to extract two alternative definitions of disorder and one definition of linear interacting peptides. The first disorder definition is based on the pLDDT score which is a per-residue estimate of the prediction accuracy. In MobiDB residues with a pLDDT lower than 70% are considered disordered. The second definition of disorder is provided by the per-residue relative solvent accessibility (RSA) of the predicted structure, as provided by the DSSP software. The RSA is averaged on a sliding window of 25 residues and positions with an average RSA over 0.58 are considered disordered. The two definitions provide similar results but are complementary at the same time. For example, there are well folded secondary structure elements, e.g. alpha helices, which can be predicted with high confidence (high pLDDT) and at the same time be found inside an extended loopy region disconnected from the rest of the structure and therefore with a high RSA. These regions are likely to undergo conditional (un)folding and can be probably associated with binding events. pLDDT and RSA are therefore combined to also infer LIPs. Figure 2 shows an example of conditional folding in the human Paxillin protein (MobiDB accession: P49023). The predicted LIPs match exactly five conserved leucine-rich (LD) motifs that interact with a variety of focal adhesion proteins as shown in different X-ray and NMR experiments (39,40). An additional LIP, region 46–52, interacts with the SH3 domain of Ponsin (41) and another region, 115–120, forms a beta sheet with a distant globular fragment of the protein itself. The AlphaFold-disorder script is available for download at URL: https://github.com/BioComputingUP/AlphaFold-disorder. In MobiDB, AlphaFold structures are downloaded from AlphaFoldDB (42). At the time of writing, all Swis-Prot and model organism proteins are processed and stored in the database, for a total of 1 121 068 entries.

### Sequence conservation

Manually curated entries are enriched with evolutionary information at the sequence level. Conservation is expressed with the height of the consensus character derived from the logo calculation (43). The logo is calculated from a multiple sequence alignment (MSA) generated from a PSI-BLAST search against the reference proteomes database clustered at 15% identity (44). The aligned sequences are combined in a single MSA by simply trimming columns corresponding to gaps in the input sequence. Given the low redundancy and diversity of the sequence database and the high chance of gaps in the MSA, in particular at the sequence ends, we also provide column occupancy indicating the percentage of non-gap rows for each column of the MSA.

### Binding modes

Molecular interactions have a particular significance for IDPs. The structural properties and the amino acid composition of disordered interacting interfaces provides a set of binding modes which are completely different from the canonical lock-and-key mechanism of well structured partners. IDP interactions are mainly provided by electrostatic forces and are entropy-driven resulting in the formation of fuzzy complexes (45). Intrinsically disordered regions (IDRs) can undergo disorder-to-order transitions and fold upon binding, or remain disordered in a partner-bound form (23). The folding energy of the interaction is compensated by an increase of structural heterogeneity of the rest of the protein. Binding modes of disordered regions refer to the conformational transitions of IDRs upon interacting with specific partners. Some IDRs exhibit context-dependent binding with different partners or cellular conditions (46). MobiDB aims at collecting as much evidence as possible about the location of binding IDRs in the sequence and about their binding modes.

MobiDB provides in the entry page an entire section dedicated to IDRs. Binding IDRs in MobiDB are called linear interacting peptides (LIPs), referring to their extended conformation. Similarly to disorder evidence, in MobiDB there are different levels of evidence (annotation confidence) and different features, the binding modes, that can be associated with a LIP. In Table 3 are shown the types of annotations currently in MobiDB. Whenever available the binding mode is provided, otherwise the region is annotated simply as a LIP. Despite curated databases capturing different binding specificity or subclass, e.g. the Eukaryotic Linear Motif (ELM) database annotates short linear motifs (SLiMs) (33), only FuzDB annotations are associated with a binding mode. FuzDB describes ‘fuzzy’ complexes that remain disordered in the bound state, ‘disorder-to-disorder’ transitions. Other annotations relative to binding modes are provided by an internal pipeline that derives this information from PDB structures by comparing disordered residues in free and bound form, as described in (23), and using the RING software to detect intermolecular interactions (16). PDB complexes are also processed by the FLIPPER (37) classifier that extracts generic LIP annotations looking at the geometrical and physicochemical properties of the structure. Large scale LIPs predictions from sequence are provided by ANCHOR (47), while AlphaFold structures are used to derive LIPs likely to be associated with conditional folding and binding modes (see AlphaFold and conditional disorder).

**Table 3.** Binding knowledge provided by MobiDB

Evidence	Feature	Source	Proteins	Description
Curated	LIP	DisProt, IDEAL	970	All LIP types
		ELM	75	Short Linear Motifs (SLiMs)
		DIBS	498	LIP interacting with structure
		MFIB	246	LIP interacting with LIP
	Binding mode	FuzDB	328	Fuzzy complexes
Derived	LIP	FLIPPER	10 728	All LIP types
	Binding mode	RING	16 606	Structural transition
Predicted	LIP	AlphaFold	991 606	Structural transition
		ANCHOR	>130 M	All LIP types

### Liquid-liquid phase separation

As for the binding modes MobiDB is committed to enrich the functional knowledge and the biological role of IDPs. The formation of dynamic liquid droplets and the phenomenon of protein phase separation are thought to be driven by disordered regions forming transient interactions (4). The new release of MobiDB integrates two databases of LLPS protein drivers, PhaSePro (36) and PhaSepDB (27). These are the only two databases that specify the regions that mediate this process. Although the general biological roles of phase separation remain to be elucidated, systematic annotation of regions undergoing LLPS will contribute to elucidating the underlying sequence-codes.

## IMPLEMENTATION

### Data generation and updates

The MobiDB database schema has now reached its maturity and has proved to be effective and fast in serving data, allowing complex queries. The last MobiDB release has focused on the simplification and acceleration of content updates. Now the entries are splitted into two different collections depending on their annotation level (see MobiDB annotation pyramid). The subset of entries with ‘curated’, ‘derived’ or ‘homologous’ annotations, and those that are extracted from AlphaFoldDB entries are stored in a so-called ‘gold’ collection. The rest of the proteins are annotated only with MobiDB-lite predictions. The ‘gold’ collection is relatively small (1.5 millions proteins) and it is regenerated from scratch at each release update. The data is processed automatically through a workflow that takes a couple of weeks of calculation. All annotations integrated from member databases undergo stringent sanity checks that verify sequences and identifiers and ‘out-of-index’ regions. MobiDB uses UniProtKB as the reference for protein identifiers and sequences. When the member database sequence does not match the sequence provided by UniProtKB for that identifier, the annotation is discarded, meaning the member database has not been updated.

The separation of entries into different collections is completely transparent to the user since database queries are always issued on both collections and results are combined at the server level.

### Controlled vocabularies

Given the amount of different annotation sources and processing procedures employed, the new MobiDB release provides a controlled vocabulary (CV) that is used as a reference to describe precisely the different annotation features. Most of the current terms in the CV were already used in the previous release, now they are fully described and clearly exposed in the website. CV terms are split into three main categories, or name spaces: (i) evidence, (ii) feature and (iii) source of information. The evidence namespace represents the different levels of the MobiDB pyramid. Feature terms represent the type (or flavor) of the annotation, while the source can be a database, a piece of software or in general provide information about the method used to generate that annotation. All annotations in MobiDB are fully identified by triplets of CV terms, one for each category. Currently 4, 39 and 37 terms populate the three groups, respectively.

### Website

The MobiDB website has been renewed, in particular the entry page, to help the user appreciate and explore more easily the amount of different annotations that are provided. Other notable changes regard the integration of the AlphaFold predicted structures and a page that shows the complete controlled vocabulary used to identify and describe all types of annotations available in MobiDB. Moreover, we have improved the API documentation by implementing a Swagger UI where the user can build and try a custom query directly on the MobiDB website. The page includes documentation of all output fields and their values.

### Entry page

The entry page has been extensively refactored preserving the functionality and the style already available in the previous release. Beside the general protein information pulled from UniProtKB (gene, protein name, localization, etc.), MobiDB now provides its annotations grouped into three different tabs: i) overview, ii) disorder and iii) binding. This separation allows the user to focus on specific aspects and delve into the hierarchical structure of the provided information more easily.

As in the previous version, the central component of the entry page is the feature viewer (48), which shows the type and position of the annotated regions. The feature viewer propagates user actions (region selections and clicks) to the sequence and structure viewers, which are instances of ProSeqViewer (49) and Mol* (50) plugins, respectively. Selected regions (or tracks) are highlighted synchronously in all the viewers.

When the entry page is open for the first time, the structure viewer loads the AlphaFold prediction which is available for about all MobiDB entries. For those annotations derived from experimental PDB structures, e.g. LIPs in protein complexes or mobile residues in NMR ensembles, the structure viewer can load the corresponding PDB entry through a button placed aside the corresponding track in the feature viewer.

Other improvements regard an inset card that pops-up at the bottom of the page when a region is selected on the feature viewer. The new card provides detailed information about the region position and the origin of the carried information.

## CONCLUSIONS AND FUTURE WORK

The great accuracy recently reached by AlphaFold (24) in the prediction of structural domains and its application to the full set of known proteins (42) has opened a Pandora's box by revealing the large fraction of proteomes that received a low confidence score. These regions are predicted as extended loops and appear to be randomly placed in order to avoid interactions with the rest of the protein and to minimize the overall moleculecular volume. Indeed, these regions correlate very well with experimental disorder and with state-of-the art disorder predictors, as shown in the CAID assessment (25,26). For the community of MobiDB users, the abundance of disorder in AlphaFold predictions is not a surprise, but rather a confirmation.

The new version of MobiDB improves the focus on the increase of functional and structural knowledge about conformational ensembles, also exploiting the great added value provided by the visualization of AlphaFold structures. A new homology transfer pipeline increased the number of entries with high quality annotations by two orders of magnitude. The new design of the entry page provides a better visualization of disorder functions. The formalization of a controlled vocabulary clarifies the source, type and classification of all annotated features and improves their accessibility.

MobiDB proved to be a mature and sustainable resource given its 10 years of history. The roadmap for the coming years is to keep working on the integration, generation and standardization of meaningful disorder knowledge. Of note is the collaboration with the IDP Community (51) of ELIXIR, the European infrastructure for biological data, and the effort in reaching out complementary deposition databases, namely the Biological Magnetic Resonance Data Bank (BMRB) (52), Small Angle Scattering Biological Data Bank (SASBDB) (53), Protein Circular Dichroism Data Bank (PCDDB) (54) Protein Ensemble (PED) (55) databases, in order to identify a standard exchange format to support disorder evidence. MobiDB is part of the IDPcentral consortium by adopting BioSchemas markup (https://bioschemas.org) (56) which allows connecting MobiDB annotations with other services based on graph data.

## DATA AVAILABILITY

All the data and link to used software are available at https://mobidb.org.
